# Heterogeneous Group of Genetically Determined Auditory Neuropathy Spectrum Disorders

**DOI:** 10.3390/ijms252312554

**Published:** 2024-11-22

**Authors:** Anastasiia A. Buianova, Marina V. Bazanova, Vera A. Belova, Galit A. Ilyina, Alina F. Samitova, Anna O. Shmitko, Anna V. Balakina, Anna S. Pavlova, Oleg N. Suchalko, Dmitriy O. Korostin, Anton S. Machalov, Nikolai A. Daikhes, Denis V. Rebrikov

**Affiliations:** 1 The Center for Precision Genome Editing and Genetic Technologies for Biomedicine, Pirogov Russian National Research Medical University, 117513 Moscow, Russia; buianova_aa@rsmu.ru (A.A.B.); verusik.belova@gmail.com (V.A.B.); galit.ilyina@gmail.com (G.A.I.); alinasamitova16@gmail.com (A.F.S.); annashmi97@gmail.com (A.O.S.); pavlova.a.s@gmail.com (A.S.P.); olegsuchalko@gmail.com (O.N.S.); d.korostin@gmail.com (D.O.K.); 2 FSBI ‘The National Medical Research Center for Otorhinolaryngology of the Federal Medico-Biological Agency of Russia’, 123182 Moscow, Russia; marishkab1993@mail.ru (M.V.B.); abc2021@yandex.ru (A.V.B.); anton-machalov@mail.ru (A.S.M.); otolar@fmbamail.ru (N.A.D.); 3 Pirogov Russian National Research Medical University, 117997 Moscow, Russia; 4 FSBI ‘National Medical Research Center for Obstetrics, Gynecology, and Perinatology Named After Academician V.I. Kulakov’, 117198 Moscow, Russia

**Keywords:** sensorineural hearing loss, cochlear implant, hearing aid, pediatrics, whole-exome sequencing

## Abstract

Auditory neuropathy spectrum disorder (ANSD) is often missed by standard hearing tests, accounting for up to 10% of hearing impairments (HI) and commonly linked to variants in 23 genes. We assessed 122 children with HI, including 102 with sensorineural hearing loss (SNHL) and 20 with ANSD. SNHL patients were genotyped for common *GJB2* variants using qPCR, while ANSD patients underwent whole exome sequencing, with variants analyzed across 249 genes. Homozygous *GJB2* variants were found in 54.9% of SNHL patients. In 60% of ANSD patients, variants were detected in *OTOF* (25%), *CDH23*, *TMC1*, *COL11A1, PRPS1, TWNK,* and *HOMER2* genes, including eight novel variants. Transient evoked otoacoustic emissions testing revealed differences at 4000 Hz (*p* = 0.0084) between the ANSD and SNHL groups. The auditory steady-state response (ASSR) test showed significant differences at 500 Hz (*p* = 2.69 × 10^−4^) and 1000 Hz (*p* = 0.0255) compared to pure-tone audiometry (PTA) in ANSD patients. Our questionnaire shows that the parents of children with SNHL often report an improved quality of life with hearing aids or cochlear implants, while parents of children with ANSD frequently experience uncertainty about outcomes (*p* = 0.0026), leading to lower satisfaction.

## 1. Introduction

Auditory neuropathy spectrum disorder (ANSD) encompasses a wide range of hearing impairments (HI) of varying severity with rehabilitation outcomes rather difficult to predict [1]. The auditory signs of ANSD include preserved otoacoustic emissions (OAEs), cochlear microphonics potential (CM), and absent acoustic reflexes. In Russia and several other countries, the first stage of newborn hearing screening relies solely on OAE testing. Therefore, patients with ANSD often go undetected and are identified late. Since patients with ANSD have normal OAEs, they are not referred for the second stage of audiological screening, which results in the delayed diagnosis of ANSD [2,3,4]. The second stage of screening is for children who did not pass the newborn hearing screening or for those who have risk factors for HI. Among children with ANSD, hearing thresholds may improve in 58.7% of cases and even normalize in 35.5% by the age of 1.5 years [5]. According to the pertinent literature sources, the prevalence of ANSD varies from 1% to 10% among individuals with HI [6,7,8].

ANSD is often linked to prematurity, hyperbilirubinemia, congenital cytomegalovirus infection, and genetic disorders [1]. However, most ANSD cases are genetically determined [9]. ANSD development is associated with variants in 23 genes [10].

Based on the localization of pathology, ANSD is classified into the pre-synaptic and post-synaptic forms [11]. The genes related to the pre-synaptic form include *OTOF*, *SLC17A8*, *CACNA1D*, and *CABP2*. Variants in these genes disrupt the inner hair cell function in the inner ear, making the affected patients potential candidates for cochlear implantation (CI). Genes linked to the post-synaptic forms of ANSD include *DIAPH3*, *OPA1*, *ATP1A3*, *MPZ*, *PMP22*, *NEFL*, *TIMM8A*, *AIFM1*, and *WFS1*. Employing CI for aural rehabilitation in patients carrying the variants that cause the post-synaptic form did not prove successful [12]. *OTOF* variants predominantly cause non-syndromic ANSD [13]. Patients with OTOF-associated ANSD typically respond better to CI than to hearing aids (HA). Over 200 variants in the *OTOF* gene have been identified, with these variants accounting for congenital ANSD in more than 41% of cases in China [14].

Revealing the genetic background underlying ANSD allows for more accurate prognosis of speech development in children after CI. A review of 33 studies involving CI in children with ANSD demonstrated improvement in speech, language, and auditory parameters [15]. Predicting the efficiency of patient rehabilitation requires establishing the precise disorder etiology including an underlying genetic factor. Comparing auditory profiles among patients with different genetic variants is essential for timely diagnosis and successful auditory rehabilitation, making it a critically relevant research focus.

The aim of our study is to enhance the early-stage ANSD diagnosis efficacy through a comparative analysis of patient auditory profiles and genotype–phenotype matching.

## 2. Results

### 2.1. Molecular Genetic Testing and the Genotype-Phenotype Matching Analysis

**Figure 1:**
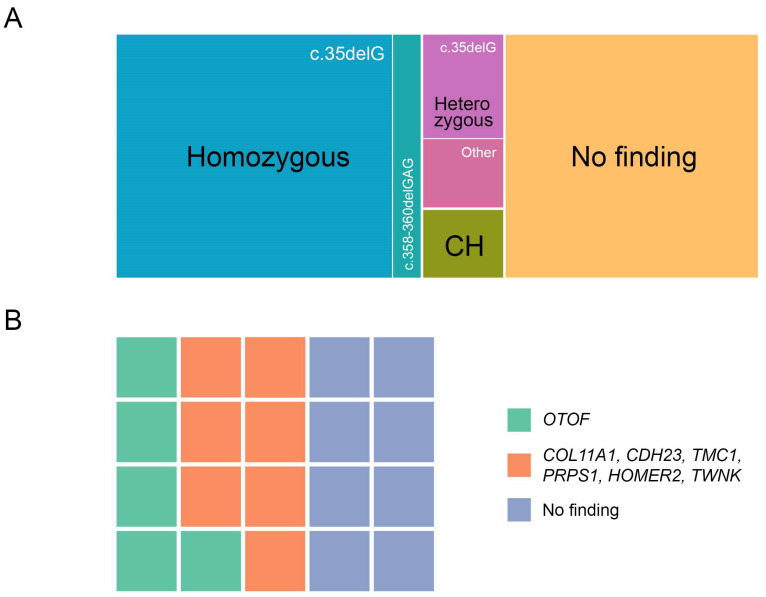
The distribution of patients depending on the presence of variants in different genes. (**A**) the investigated groups include conventional sensorineural hearing loss and (**B**) auditory neuropathy spectrum disorders. Abbreviations: CH—compound heterozygote.

As shown in Figure 1, genetic variants were observed in most patients. In our study, 56 (54.90%) sensorineural hearing loss (SNHL) patients had homozygous (47—c.35delG, 5—c.358–360delGAG, total 50.98%) and compound heterozygous (3—c.35delG/c.313–326del14, 1—c.35del/c.358–360delGAG, 3.92% in total) *GJB2* variants. Additionally, 12 (60%) ANSD patients presented with nucleotide sequence variants in various genes. Furthermore, we identified 10 carriers of *GJB2* gene variants, including 6 patients with c.35delG, and 2 each with c.358–360delGAG and c.313–326-del14 variants.

The children with ANSD were included in the group I and the children with conventional SNHL were included in group II, respectively. Molecular genetic testing demonstrated that the prevalence of the genetically determined hearing loss among the children undergoing treatment in the FSBI ‘The National Medical Research Center for Otorhinolaryngology of the Federal Medico-Biological Agency of Russia’ was 55.74%.

Analyzing the data from the children with ANSDs, we noted high levels of heterogeneity of this disorder (Table 1). Nevertheless, the pathogenic variants in the *OTOF* gene were most frequently observed, occurring in homozygous or compound heterozygous states in five out of twenty patients.

The extended survey results are presented in Table S1. This table includes two carriers of clinically significant variants in genes associated with ANSD, as follows: the boy with CI carried both *OTOF*(NM_194248.3): c.2165G > C (p.Arg722Pro) and *SPNS2*(NM_001124758.3): c.310G > C (p.Ala104Pro) variants, while the girl with HA carried the *CDH23*(NM_022124.6): c.5386C > A (p.Pro1796Thr) variant. All three variants have not been previously described.

Among the children with confirmed diagnoses, we identified eight nucleotide sequence variants that have not been previously reported in the literature. We classified the following variants as likely pathogenic: *OTOF*(NM_194248.3): c.3021G > C (p.Trp1007Cys) (CADD score = 31), *TMC1*(NM_138691.3): c.1592A > T (p.Asp531Val) (CADD score = 31), *TMC1*(NM_138691.3): c.421_425del (p.Arg141ValfsTer4), *TWNK*(NM_021830.5): c.561_562insA (p.Asp188ArgfsTer38), *PRPS1*(NM_002764.4): c.202A > G (p.Met68Val) (CADD score = 23). The data from the literature reported the patient with the Charcot–Marie–Tooth disease and bilateral SNHL onset at 6–10 years of age; this patient had an altered amino acid sequence at the same position (p.Met68Leu) as identified in our patient no. 12 [21]. We classified the following variants as having uncertain clinical significance: *COL11A1*(NM_001854.4): c.1678C > T (p.Pro560Ser) (CADD score = 28.5), *HOMER2*(NM_004839.4): c.992A > C (p.Asp331Ala) (CADD score = 27.4), *TWNK*(NM_021830.5): c.1852C > T (p.Pro618Ser) (CADD score = 22.5). Despite the p.Pro618Ser variant being predicted as benign, it is located in the functional SF4 helicase domain, which contains 116 missense/in-frame variants, including 36 pathogenic variants, 79 variants of unknown significance, and only 1 benign variant.

Pathogenic variants in the *OTOF* gene were transmitted from mothers to patients 4 and 5 (fathers were not available for genetic analysis). Patient 9 inherited the *TMC1* p.Arg141ValfsTer4 and p.Asp531Val variants from his mother and father, respectively. Patient 12 inherited the *PRPS1* variant p.Met68Val from his mother.

Patient 5 also harbored a previously undescribed variant *TECTA*(NM_005422.4): c.4966A > G (p.Met1656Val) in a heterozygous state, associated with deafness, autosomal dominant 8/12 (MIM:601543), and deafness, autosomal recessive 21 (MIM:603629). However, according to CADD, this variant was classified as benign (score = 22.6).

Patient 11 was monitored throughout infancy for breath-holding spells and perinatal brain injury. The patient was diagnosed with delayed speech development. Five years after the onset of the condition, the hearing loss was observed, initially unilateral and later bilateral. The patient is under the care of an otorhinolaryngologist and audiologist with a diagnosis of stage 2 bilateral mixed (predominantly sensorineural) hearing loss. Additionally, mild myotonic syndrome was diagnosed by a neurologist. Stage 2 bilateral SNHL is present, along with speech impairment, asthenic syndrome, and behavioral issues. A psychiatrist diagnosed the patient with F06.7 (ICD-10).

### 2.2. Hearing Test Results

All parents of patients in groups I and II reported hearing loss (100%), with 44% also reporting speech delays (N = 54). No other complaints were noted by the patients or their legal representatives.

Comprehensive otolaryngological examinations confirmed that none of the children had acute ENT pathology at the time of assessment. Additionally, computed tomography (CT) scans of the temporal bones and magnetic resonance imaging (MRI) of the brain revealed no pathological changes.

**Figure 2:**
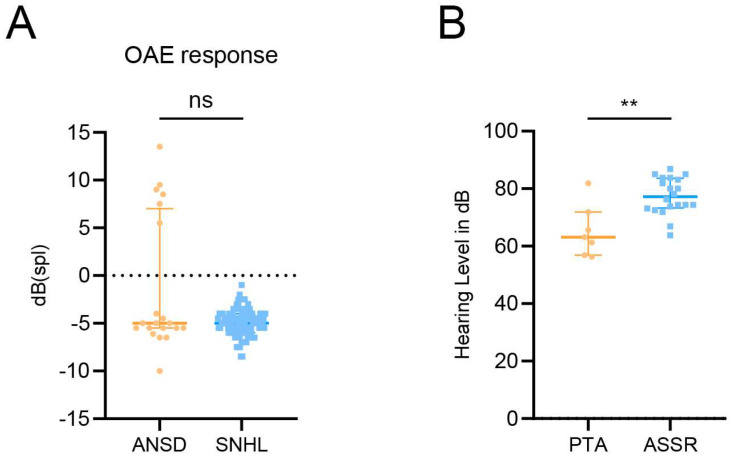
Comprehensive audiologic assessments. (**A**) comparative analysis of mean signal-to-noise ratios during transient-evoked otoacoustic emissions (TEOAEs) registration in patients from the group I (auditory neuropathy spectrum disorders, ANSDs) and group II (sensorineural hearing loss, SNHL). (**B**) comparison of mean results from pure-tone audiometry (PTA) and auditory steady-state response (ASSR) tests in patients with ANSDs. Abbreviations: OAEs—otoacoustic emissions. Mann–Whitney U test. *p* < 0.01 is designated as ‘**’, ns—not significant (*p* > 0.5).

In our study, a comparative analysis of the signal-to-noise ratio during the registration of transient evoked otoacoustic emissions (TEOAEs) (Figure 2A, Table 2) revealed a statistically significant difference at 4000 Hz (*p* = 0.008348) between the ANSD and SNHL patients. The least pronounced difference was observed at 500 Hz, since both groups showed negative signal-to-noise ratios. However, a comparison of the mean values across all frequencies did not reveal any statistically significant differences (*p* = 0.5633).

**Figure A1:**
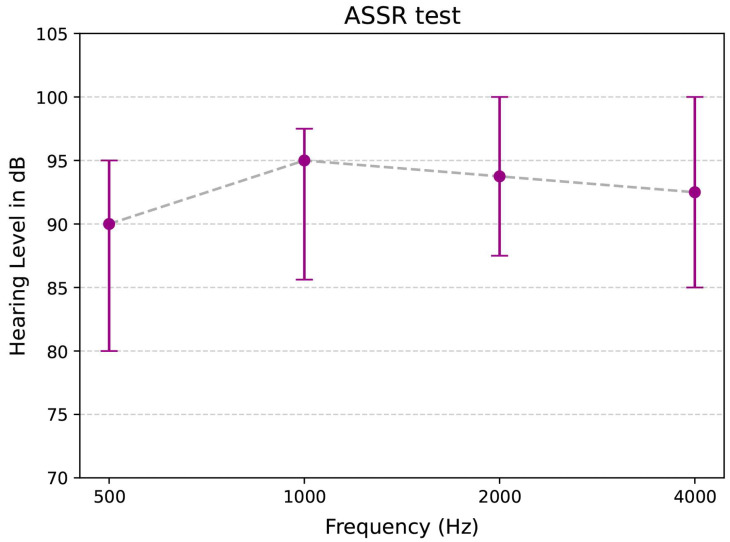
Auditory steady-state response (ASSR) test values in 94 patients from group II (sensorineural hearing loss, SNHL) with severe and profound hearing loss. The graph displays the median values along with the interquartile range (Q1-Q3) for each frequency.

Table A1 presents the median and interquartile range (Q1-Q3) of the auditory steady-state response (ASSR) test results in patients from Group II, with values separately for the right and left ears at each frequency. The data in Figure A1 correspond to patients with severe and profound hearing loss (N = 94), for whom CI was indicated.

In ANSD patients, results from the pure-tone audiometry (PTA) test indicated lower thresholds compared to those recorded during the ASSR test (*p* = 0.009585) (Figure 2B). Comparative analysis of the metrics obtained from the ASSR and PTA tests revealed statistically significant differences at frequencies of 500 Hz (*p* = 0.0002689) and 1000 Hz (*p* = 0.02546) within the ANSD patient group (Table 3). However, during this study, not all patients were able to perform PTA due to the age of the patients or lack of understanding of the task.

Acoustic impedance testing revealed that all patients exhibited a type ‘A’ tympanometric curve, as classified by Jerger (1970) [22]. In all examined children with ANSDs, as well as those with SNHL of the IV degree, the acoustic reflex was absent. Auditory brainstem response (ABR) testing with click stimuli showed that in patients with ANSDs (group I), the V wave peak was undetectable bilaterally, though the CM (I wave) was measured. Specifically, the CM was detected at an 80 dB stimulus in 40% (eight patients) and at 90 dB in 60% (twelve patients). Among the SNHL patients (group II), the V wave peak was observed primarily at 90–100 dB in 94 cases, at 40 dB in one child, and at 60–70 dB in four patients.

The degree of hearing loss in ANSD patients was determined based on subjective hearing assessment, whereas for SNHL patients, the degree of hearing loss was established using objective methods such as ASSR and ABR tests.

**Figure 3:**
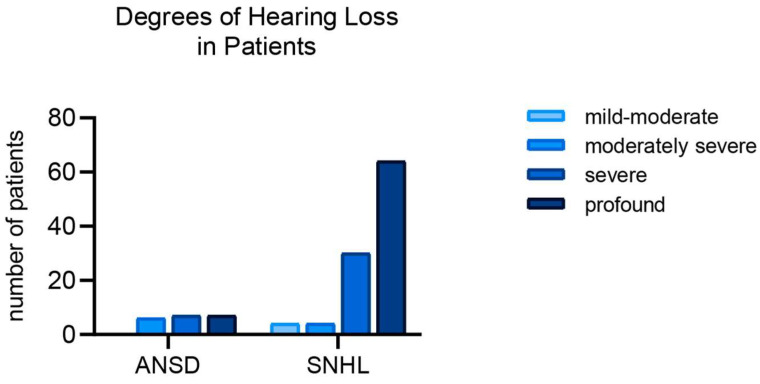
Distribution of patients with auditory neuropathy spectrum disorders (ANSDs) and sensorineural hearing loss (SNHL) by degree of hearing loss (mild—26–40 dB, moderate—41–55 dB, moderately severe—56–70 dB, severe—71–90 dB, profound—91+).

Figure 3 illustrates the distribution of hearing loss severity among patients with ANSDs and SNHL. In patients with ANSDs, a more severe degree of hearing loss predominates (seven cases of ‘severe’ and seven of ‘profound’), and there are no cases of mild or moderate hearing loss. Patients with SNHL are represented across all severity categories, with a significant portion in the ‘severe’ and ‘profound’ categories (30 and 64, respectively). The Pearson’s chi-square test was applied, revealing a statistically significant relationship between the type of hearing loss (ANSDs or SNHL) and the degree of hearing loss (χ² = 17.04, df = 3, *p* = 0.00069). Post hoc analysis with a Bonferroni correction showed that significant differences were observed in the following severity categories: moderate (*p* = 0.017) and profound (*p* = 0.002) hearing loss, where SNHL was found to exhibit a tendency towards a greater severity of loss compared to ANSDs. We cannot consider the results of the chi-square test valid, as the conditions were not met—all expected values should be greater than 1, and at least 20% of the expected values should exceed 5. Therefore, we applied Fisher’s exact test, which is used for small sample sizes or when expected frequencies are low. We calculated the *p*-values for each comparison using 2 × 2 contingency tables for each pair of severity categories and applied a Bonferroni correction. The only statistically significant comparison was between moderately severe and profound hearing loss, with a *p* = 0.0008 and an adjusted *p* = 0.0047. This indicates that there is a significant difference between the severity of hearing loss in these categories for patients with ANSDs and SNHL, with SNHL showing a greater severity of hearing loss in these two categories compared to ANSDs.

### 2.3. Quality of Life Assessment

Patients were stratified based on the use of the following rehabilitation tools: 11 ANSD patients and 83 SNHL patients used CI, while 9 ANSD patients and 19 SNHL patients used HAs. In both groups, patients predominantly used CI.

According to the survey (Document S1), SNHL patients had been using technical devices longer (20.37 ± 16.4 months) than ANSD patients (14.35 ± 13.1 months).

**Figure 4:**
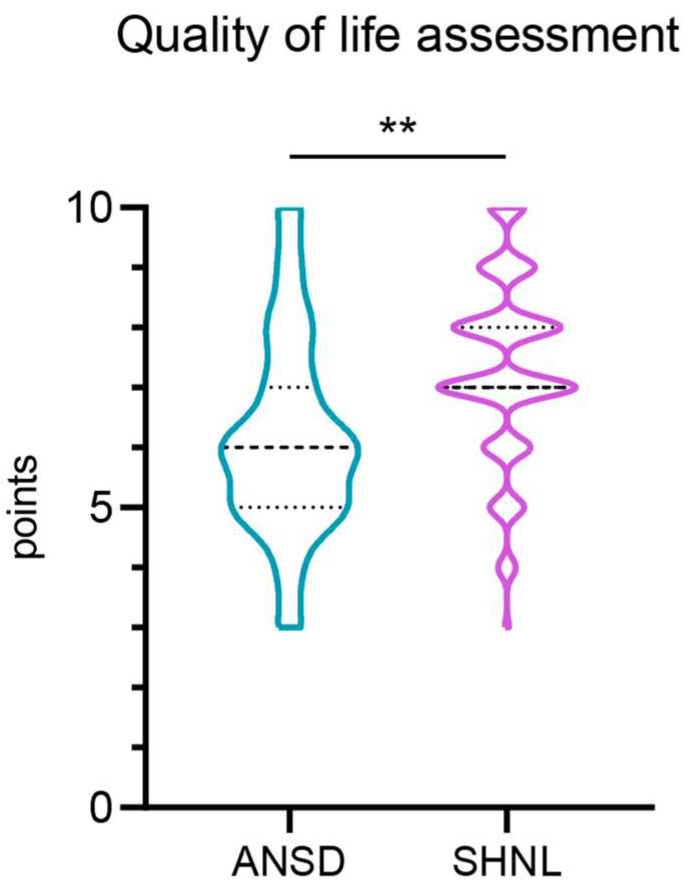
Results of assessing changes in the quality of life of children with auditory neuropathy spectrum disorders (ANSDs) and sensorineural hearing loss (SNHL) on a 10-point scale (0—life has not changed, 10—life has become brighter and more fulfilling). Mann–Whitney U test. *p* < 0.01 is designated as ‘**’.

Changes in the quality of life since the start of HA or CI use, presented on a 10-point scale in Figure 4, indicate that the parents of children with SNHL are generally more satisfied with their children’s improvements than parents of children with ANSDs (*p* = 0.0026). On a 10-point scale, parents of children with SNHL reported a median satisfaction score of 7 [9,10], whereas parents of children with ANSDs reported a median satisfaction score of 6 [7,9].

## 3. Discussion

In our study, the genetically determined hearing loss accounted for 74% of all children undergoing both treatment and monitoring at the FSBI ‘The National Medical Research Center for Otorhinolaryngology’ of the Federal Medico-Biological Agency of Russia, with ANSD patients comprising 17.65% of these cases. The most common genetic cause of ANSDs observed was variants in the *OTOF* gene, consistent with the existing literature, occurring in 25% of cases in our study.

The observed improvement in hearing in children with ANSDs following gene therapy represents a promising advancement in the treatment of genetic hearing loss. While CI continues to be the primary method for auditory and verbal rehabilitation in ANSD patients, gene therapy offers a potentially more effective and natural alternative. Gene therapy using AAV1-hOTOF for bilateral hearing loss in five children showed no serious adverse effects and improved hearing and speech perception. The average auditory brainstem response threshold was restored from >95 dB to 50–85 dB, and all patients recovered speech perception and sound localization ability [23].

From our findings, only a subset of the identified genes (*OTOF*, *TWNK*, *PRPS1*) could be directly linked to ANSDs. For instance, one patient with compound heterozygosity for the p.Arg1583His and p.Gln1883* variants in the *OTOF* gene underwent cochlear implantation at the age of 20 months. After five years of monitoring, their average pure tone thresholds at 500, 1000, 2000, and 4000 Hz increased by 25–37.5 dB [24]. Another patient with Perrault syndrome, associated with *TWNK* variants and onset of hearing loss at 5.5 years, received a CI at 6.5 years old. After 1 year, the average hearing threshold increased from 98.75 dB to 38.25 dB; the vowel, consonant, disyllable, and tone recognition scores in the quiet field were 36, 36, 36, and 56%, respectively [25]. In this patient and another patient carrying compound heterozygous variants in the *TWNK* gene, CI improved CAP and SIR scores.

The contribution of the *COL11A1*, *CDH23*, *TMC1*, and *HOMER2* genes to ANSD development has not been previously described, which emphasizes that we still lack profound knowledge of this disease. CI was found to be completely ineffective for a German patient with the heterozygous variant *COL11A1*(NM_080629.2): c.2644C > T (p.Arg882Trp) [26]. Conversely, patients with *CDH23* variants demonstrated improvements in hearing and speech outcomes post-CI [27], including those with the *CDH23*(NM_022124.6): c.2591G > T (p.Gly864Val) variant in a compound heterozygous state, similar to findings in patient 6 [18]. Although the *CDH23*(NM_022124.6): c.6442G > A (p.Asp2148Asn) variant is prevalent in Europe with 138 healthy heterozygous individuals reported [28], no cases of CI in patients with this variant have been documented. Excellent clinical outcomes were observed with CI in two half-siblings with compound heterozygosity at the *TMC1* gene (p.Arg34 and p.Trp321*) and in a patient with variants p.Arg389* and p.Arg512* [29]. *HOMER2*-associated deafness is extremely rare, with the only described variants being p.Arg196Pro, p.Met281Hisfs*9, and p.Pro278Alafs*10. The patients carrying these variants did not undergo CI [30]. As whole genome and whole exome sequencing become more accessible, the development of multigene panels, including ‘hearing loss’ panels with dozens of genes associated with hearing loss, presents a promising approach for detecting rare forms of congenital hearing loss in ANSD patients.

Audiological testing is essential for diagnosing ANSDs and encompasses a range of hearing assessment methods.

TEOAEs revealed a statistically significant difference between ANSDs and SNHL patients at 4000 Hz. Notably, patients with *OTOF* gene variants continued to exhibit TEOAE responses regardless of age, consistent with the existing literature [31]. In contrast, TEOAEs were absent in other patients, and the signal-to-noise ratio was negative, leading to no significant differences between the groups.

Acoustic impedance testing showed that all ANSD patients in our study had a type ‘A’ tympanometric curve, with no acoustic reflexes recorded. In ABR testing with click stimuli, CM in ANSD patients were typically detected at 80 and 90 dB.

Given the clinical and audiological characteristics of ANSDs, we conducted a statistical analysis comparing the results of PTA testing with visual reinforcement and the ASSR test. This analysis revealed a significant difference only at 500 Hz and 1000 Hz. Due to age and difficulty comprehending instructions, not all patients were able to complete behavioral audiometry to determine behavioral thresholds. Survey responses from parents of children with sensorineural hearing loss commonly indicated positive quality-of-life improvements after using HA or CI. Conversely, parents of children with ANSDs may face greater uncertainty in the prognosis of the child’s development, which leads to a low level of satisfaction with the quality of life.

Currently, it is not possible to draw comprehensive conclusions on auditory and speech rehabilitation outcomes for ANSD patients, as not all have reached the 5-year milestone of HA or CI use. As we expand the cohort of patients with genetically confirmed ANSDs and extend the observation period, we will be able to assess the long-term effectiveness of CI and HA in this population. This ongoing research aims to refine the criteria for cochlear implantation and develop an optimized rehabilitation algorithm tailored for children with ANSDs.

## 4. Materials and Methods

### 4.1. Patients

This study was conducted from 2021 to 2024 at the clinical base of the FSBI ‘The National Medical Research Center for Otorhinolaryngology’ of the Federal Medico-Biological Agency of Russia, as well as in the laboratories of the Russian National of Further Professional Education, the Russian Medical Academy of Continuous Professional Education, and the FSBI ‘National Medical Research Center For Obstetrics, Gynecology, And Perinatology Named After Academician V.I. Kulakov’. All patients provided written informed consent for the sample collection, subsequent analysis, and publication thereof.

We examined 122 children with HI, including 102 pediatric patients (mean age: 3.7 ± 4.1 years) with various degrees of SNHL and 20 children with clinically confirmed ANSDs (mean age: 5.65 ± 4.63 years).

All children underwent comprehensive clinical and audiologic assessments, which included the collection of complaints and case history, a complete otolaryngological examination, CT imaging of the temporal bones, and MRI of the brain. The audiological evaluations involved the following procedures: acoustic impedance testing (Interacoustics AT235; Interacoustics A/S Drejervaenget 8 DK-5610, Assens, Denmark), recording TEOAEs (Interacoustics Eclipse EP25; Interacoustics A/S Drejervaenget 8 DK-5610, Assens, Denmark) and short-latency auditory evoked potentials (SLAEPs) using Chirp-LS signals (Interacoustics Eclipse EP25; Interacoustics A/S Drejervaenget 8 DK-5610, Assens, Denmark), as well as in children with ANSDs using click signals with stimuli of different polarities (CM registration) (Interacoustics Eclipse EP25; Interacoustics A/S Drejervaenget 8 DK-5610, Assens, Denmark). For ANSD patients, auditory evaluations involved recording click signals in response to stimuli of different polarities (rarefaction and condensation phases). Additionally, an ASSR test was performed (Interacoustics Eclipse EP25; Interacoustics A/S Drejervaenget 8 DK-5610, Assens, Denmark) and the children with ANSDs also underwent PTA testing (Interacoustics AC40; Interacoustics A/S Drejervaenget 8 DK-5610, Assens, Denmark).

ANSDs were identified based on the following audiological signs: lack of acoustic reflex registration, presence of normal CM, presence or absence of OAEs (depending on the timing of diagnosis), and abnormal ABR waveforms. Exclusion criteria included conductive and mixed hearing loss; chronic middle ear conditions; malformations of the external, middle, or inner ear; acute upper respiratory infections, and any history or presence of clinically significant uncontrolled diseases in any organ system.

The parents of all examined children completed the survey created by the specialists of the FSBI ‘The National Medical Research Center for Otorhinolaryngology of the Federal Medico-Biological Agency of Russia’ to assess the effectiveness of aural rehabilitation (Document S1). The survey assessed patient demographics, comfort and side effects of device use, rehabilitation support, and overall impact on quality of life, providing a comprehensive view of each child’s experience with their hearing rehabilitation device.

After the diagnosis had been established by routine hearing tests, all patients underwent molecular genetics testing.

### 4.2. qPCR

For SNHL patients, we performed genotyping of the frequent *GJB2* variants (c.35delG, c.167delT, c.235delC, c.313–326del14, and c.358–360delGAG) using the ‘Surdogenetic’ kit (JSC DNA-Technology, Moscow, Russia) following the manufacturer’s instructions using the DT-96 thermocycler (JSC DNA-Technology, Moscow, Russia).

### 4.3. Whole Exome Sequencing (WES)

For children with a clinically confirmed ANSD, we carried out WES with subsequent analysis and data interpretation.

DNA-libraries were prepared using 500 ng of genomic DNA with the MGIEasy Universal DNA Library Prep Set (MGI Tech, Shenzhen, China), following the manufacturer’s protocol. DNA fragmentation was performed via ultrasonication using Covaris S-220 (Covaris, Inc., Woburn, MA, USA) resulting in the average fragment length of 250 bp. Prior to DNA fragmentation, the libraries were pooled according to the protocol described in [32] using the SureSelect Human All Exon v7 and v8 probes (Agilent Technologies, Santa Clara, CA, USA), which cover the whole human exome. DNA and library concentrations were measured with Qubit Flex (Life Technologies, Carlsbad, CA, USA) using the dsDNA HS Assay Kit (Invitrogen, Waltham, MA, USA), following the manufacturer’s instructions. The quality of the prepared libraries was assessed using Bioanalyzer 2100 with the High Sensitivity DNA kit (Agilent Technologies, Santa Clara, CA, USA), as per the manufacturer’s protocol. Subsequently, the libraries were circularized and sequenced in the paired-end mode using the DNBSEQ-G400 with the DNBSEQ-G400RS High-throughput Sequencing Set PE100 (MGI Tech, Shenzhen, China) achieving an average coverage of 100×.

FastQ files were generated with the basecallLite software (ver. 1.0.7.84) from the manufacturer (MGI Tech, Shenzhen, China). The quality of the obtained sequencing data was assessed using the FastQC v0.11.9 software (Babraham Institute, Cambridge, UK) [33]. Based on the quality control results, the correction of raw reads was performed using the bbduk v38.96 software [34]. For each sample, we conducted the bioinformatics analysis of sequencing data which included aligning reads to the human reference genome GRCh38 with bwa-mem2 v2.2.1 (Wellcome Trust Sanger Institute, Cambridge, UK) [35] and SAMtools v1.9 (Wellcome Sanger Institute, Hinxton, UK) [36], identification of duplicates and obtaining the exome enrichment quality metrics using Picard v2.22.4 (Broad Institute, Cambridge, MA, USA) [37], variant calling using bcftools v1.9 (Wellcome Sanger Institute, Hinxton, UK) [38] and Deepvariant v1.5.0 [39], variant annotation using AnnoVar v2020Jun08 (Center for Applied Genomics, Children’s Hospital of Philadelphia, Philadelphia, PA, USA) [40], Intervar v2.2.2 (Wang Genomic Lab, Philadelphia, PA, USA) [41], and our custom Python3 scripts for the optimization and quality improvement of the final annotation files. A CNV search was performed using CNVkit v0.9.8 [42], and CNV annotation was performed with ClassifyCNV v1.1.1 (Genotek Ltd., Moscow, Russia) [43] and AnnotSV v3.2.3 [44]. After the bioinformatics analysis, we performed a final quality check with MultiQC v1.16 [45].

For this study, we assembled a panel comprising 249 genes (Table S2) associated with the diagnoses of ‘auditory neuropathy’ and ‘hearing loss’. The selection of genes was based on the Human Phenotype Ontology panels (HPO) ‘Infantile sensorineural hearing impairment (HP:0008610)’ and ‘Congenital sensorineural hearing impairment (HP:0008527)’ [46], as well as keyword searches for ‘sensorineural hearing loss’ and ‘deafness’ in the Online Mendelian Inheritance in Man (OMIM). We excluded those variants associated only with conductive hearing loss. The clinical significance of identified variants was interpreted following the ACMG criteria [47], utilizing variant databases and the literature sources. The population frequencies were obtained from gnomAD v.4.0.0 [28] и RUSeq [48].

Statistical data analysis was performed using the RStudio version 2024.03.0 (Posit PBC, Boston, MA, USA). Data with a normal distribution are presented as the mean ± SD, while non-normally distributed data are presented as the median [Q1, Q3]. The Mann–Whitney U test was used to compare quantitative data between the two groups. A *p*-value < 0.05 was considered statistically significant.

## 5. Conclusions

Our study identified statistically significant differences between ANSDs and SNHL patients in signal-to-noise ratio measures. Additionally, all patients with homozygous or compound heterozygous mutations in the *OTOF* gene (five out of twenty) exhibited transient evoked otoacoustic emissions (TEOAEs). Comparative analysis between hearing thresholds from behavioral audiometry and the ASSR test revealed statistically significant differences only at 500 Hz and 1000 Hz. At this stage, comprehensive conclusions on auditory and speech rehabilitation outcomes in ANSD patients are premature due to the limited duration of HA/CI use (less than 5 years in many cases). Further results will be shared as this study progresses.

## Figures and Tables

**Table 1:** Nucleotide sequence variants in different genes among patients with auditory neuropathy spectrum disorders (ANSDs) (N = 12, 60% of all ANSD cases in the present study).

Patient	Sex	Hearing Aids (HA)/Cochlear Implant (CI), Age at the Moment of Installation	Lifetime of HA/CI (Months)	Quality of Life Assessment (0 Points—No Change, 10 Points—Life Has Become More Fulfilling)	Genetic Variant	OMIM Disease	Reported
1	Male	HA, 16 years	4	5	*OTOF*(NM_194248.3):c.3021G > C (p.Trp1007Cys) (heterozygous)	Deafness, autosomal recessive 9 (MIM:601071)	This study
					*OTOF*(NM_194248.3): c.4747C > T (p.Arg1583Cys) (heterozygous)		[14]
2	Male	CI, 15 years	4	5	*OTOF*(NM_194248.3): c.3021G > C (p.Trp1007Cys) (heterozygous)		This study
					*OTOF*(NM_194248.3): c.4747C > T (p.Arg1583Cys) (heterozygous)		[14]
3	Male	HA, 8 years	4	5	*OTOF*(NM_194248.3): c.3021G > C (p.Trp1007Cys) (heterozygous)		This study
					*OTOF*(NM_194248.3): c.4747C > T (p.Arg1583Cys) (heterozygous)		[14]
4	Female	CI, 4 years	6	6	*OTOF*(NM_194248.3): c.4903A > T (p.Arg1635Ter) (homozygous)		[16]
5	Female	CI, 4 years	3	7	*OTOF*(NM_194248.3): c.1111G > C (p.Gly371Arg) (homozygous)		[17]
6	Female	CI, 2 years	74	10	*CDH23*(NM_022124.6): c.3067G > A (p.Asp1023Asn) (heterozygous)	Deafness, autosomal recessive 12 (MIM:601386)	[18]
					*CDH23*(NM_022124.6): c.6442G > A (p.Asp2148Asn) (homozygous)		[19]
7	Female	CI, 2 years	12	6	*CDH23*(NM_022124.6): c.6442G > A (p.Asp2148Asn) (homozygous)		[20]
8	Female	HA, 5 years	13	8	*COL11A1*(NM_001854.4): c.1678C > T (p.Pro560Ser) (heterozygous)	Deafness, autosomal dominant 37 (MIM:618533)	This study
9	Male	CI, 1 year	6	9	*TMC1*(NM_138691.3): c.421_425del (p.Arg141ValfsTer4) (heterozygous)	Deafness, autosomal recessive 7 (MIM:600974)	This study
					*TMC1*(NM_138691.3): c.1592A > T (p.Asp531Val) (heterozygous)		This study
10	Male	CI, 6 years	10	6	*HOMER2*(NM_004839.4): c.992A > C (p.Asp331Ala) (heterozygous)	Deafness, autosomal dominant 68 (MIM:616707)	This study
11	Male	HA, 14 years	8	8	*TWNK*(NM_021830.5): c.561_562insA (p.Asp188ArgfsTer38) (heterozygous)	Mitochondrial DNA depletion syndrome 7 (hepatocerebral type) (MIM:271245), Perrault syndrome 5 (MIM:616138)	This study
					*TWNK*(NM_021830.5): c.1852C > T (p.Pro618Ser) (heterozygous)		This study
12	Male	HA, 2 years	2	3	*PRPS1*(NM_002764.4): c.202A > G (p.Met68Val) (hemizygous)	Deafness, X-linked 1 (MIM:304500) Arts syndrome (MIM:301835) Charcot-Marie-Tooth disease, X-linked recessive, 5 (MIM:311070) Phosphoribosyl pyrophosphate synthetase superactivity (MIM:300661)	This study

Notes: Patients 1, 2, and 3 are siblings. CI—cochlear implant; HA—hearing aid.

**Table 2:** Comparative characterization of signal-to-noise ratios during the registration of transient evoked otoacoustic emissions (TEOAEs) in patients with auditory neuropathy spectrum disorders (ANSDs) (I group) and in patients with SNHL (II group). Mann–Whitney U test.

Frequency	Group I Ме [25%, 75%]	N Ears	Group II Ме [25%, 75%]	N Ears	*p*-Value
500 Hz	−6.25 [−7.5, −4.625]	20	−4.5 [−6, −3.5]	102	0.05552
1000 Hz	−4.725 [−6.125, 4.912]	20	−5 [−6, −3.5]	102	0.3011
2000 Hz	−4 [−5.162, 7.75]	20	−5 [−6, −3.5]	102	0.08219
4000 Hz	−3.25 [−4.75, 7.75]	20	−5 [−6.5, −3.5]	102	0.008348

Notes: Me—median.

**Table 3:** Pure-tone audiometry (PTA) test and the auditory steady-state response (ASSR) test comparison of the both ears in auditory neuropathy spectrum disorders patients. Mann–Whitney U test.

Frequency	PTA Test Ме [25%, 75%]	N Ears	ASSR Test Ме [25%, 75%]	N Ears	*p*-Value
500 Hz	52.5 [50.62, 54.38]	6	77.5 [72.5, 80]	20	0.0002689
1000 Hz	62.5 [60, 70.62]	6	81.25 [75, 85]	20	0.02546
2000 Hz	71.25 [63.75, 78.75]	6	80 [75, 85]	20	0.1584
4000 Hz	76.25 [73.12, 81.25]	6	73.75 [64.38, 83.12]	20	0.669

Notes: Me—median.

## Data Availability

The sequence data are generated from patient samples and therefore are only available under restricted access.

## Supplementary Materials

The following supporting information can be downloaded at: https://www.mdpi.com/article/10.3390/ijms252312554/s1.

## Appendix A

**Table A1:** Auditory steady-state response (ASSR) test results in patients with sensorineural hearing loss (SNHL) (group II).

Frequency, Ear	Me [25%, 75%]	N Ears
500 Hz AD	90 [80–95]	102
500 Hz AS	90 [85–100]	102
1000 Hz AD	95 [85–100]	102
1000 Hz AS	95 [90–100]	102
2000 Hz AD	95 [86.25–100]	102
2000 Hz AS	90 [90–100]	102
4000 Hz AD	90 [85–100]	102
4000 Hz AS	95 [85–100]	102

Notes: Me—median; AD—right ear; AS—left ear.

## References

[1] De Siati R.D., Rosenzweig F., Gersdorff G., Gregoire A., Rombaux P., Deggouj N. (2020). Auditory Neuropathy Spectrum Disorders: From Diagnosis to Treatment: Literature Review and Case Reports. J. Clin. Med..

[2] Wroblewska-Seniuk K.E., Dabrowski P., Szyfter W., Mazela J. (2016). Universal newborn hearing screening: Methods and results, obstacles, and benefits. Pediatr. Res..

[3] Iwasa Y.-I., Nishio S.-Y., Yoishimura H., Sugaya A., Kataoka Y., Maeda Y., Kanda Y., Nagai K., Naito Y., Yamazaki H. (2021). Detailed clinical features and genotype-phenotype correlation in an *OTOF*-related hearing loss cohort in Japan. Hum. Genet..

[4] Bennett C., Yoon P., Lee M.Y., Wolfe M., Anne S., Carvalho D.S. (2023). Newborn hearing screening methodology impacts the timing of diagnosis for auditory neuropathy spectrum disorder. Am. J. Otolaryngol..

[5] Aldè M., Di Berardino F., Ambrosetti U., Barozzi S., Piatti G., Consonni D., Zanetti D., Pignataro L., Cantarella G. (2022). Hearing outcomes in preterm infants with confirmed hearing loss. Int. J. Pediatr. Otorhinolaryngol..

[6] Foerst A., Beutner D., Lang-Roth R., Huttenbrink K.-B., von Wedel H., Walger M. (2006). Prevalence of auditory neuropathy/synaptopathy in a population of children with profound hearing loss. Int. J. Pediatr. Otorhinolaryngol..

[7] Vignesh S.S., Jaya V., Muraleedharan A. (2014). Prevalence and Audiological Characteristics of Auditory Neuropathy Spectrum Disorder in Pediatric Population: A Retrospective Study. Indian J. Otolaryngol. Head Neck Surg..

[8] Penido R.C., Isaac M.L. (2015). Prevalence of auditory neuropathy spectrum disorder in an auditory health care service. Braz. J. Otorhinolaryngol..

[9] Lieu J.E.C., Kenna M., Anne S., Davidson L. (2020). Hearing Loss in Children. JAMA.

[10] Wang H., Guan L., Wu X., Guan J., Li J., Li N., Wu K., Gao Y., Bing D., Zhang J. (2024). Clinical and genetic architecture of a large cohort with auditory neuropathy. Hum. Genet..

[11] Rance G., Starr A. (2015). Pathophysiological mechanisms and functional hearing consequences of auditory neuropathy. Brain.

[12] Shearer A.E., Eppsteiner R.W., Frees K., Tejani V., Sloan-Heggen C.M., Brown C., Abbas P., Dunn C., Hansen M.R., Gantz B.J. (2017). Genetic variants in the peripheral auditory system significantly affect adult cochlear implant performance. Hear. Res..

[13] Ford C.L., Riggs W.J., Quigley T., Keifer O.P., Whitton J.P., Valayannopoulos V. (2023). The natural history, clinical outcomes, and genotype–phenotype relationship of otoferlin-related hearing loss: A systematic, quantitative literature review. Hum. Genet..

[14] Zhang Q., Han B., Lan L., Zong L., Shi W., Wang H.-Y., Xie L., Zhao C., Zhang C., Yin Z. (2016). High frequency of *OTOF* mutations in Chinese infants with congenital auditory neuropathy spectrum disorder. Clin. Genet..

[15] Sahwan M., Abdelsamad Y., Alasfoor F., Alfayez F., Binkhamis G., Nichani J. (2023). Cochlear implantation in children with auditory neuropathy spectrum disorder: An updated systematic review. Eur. Arch. Oto-Rhino-Laryngol..

[16] Lalayants M., Mironovich O., Bliznets E., Маркoва T.G., Polyakov A., Таварткиладзе G.A. (2020). OTOF-related auditory neuropathy spectrum disorder. Vestnik Otorinolaringol..

[17] Churbanov A.Y., Karafet T.M., Morozov I.V., Mikhalskaia V.Y., Zytsar M.V., Bondar A.A., Posukh O.L. (2016). Whole Exome Sequencing Reveals Homozygous Mutations in RAI1, OTOF, and SLC26A4 Genes Associated with Nonsyndromic Hearing Loss in Altaian Families (South Siberia). PLoS ONE.

[18] Chen K., Huang B., Sun J., Liang Y., Xiong G. (2021). Cochlear Implantation Outcomes in Children With *CDH23* Mutations–Associated Hearing Loss. Otolaryngol. Neck Surg..

[19] Shearer A.E., Black-Ziegelbein E.A., Hildebrand M.S., Eppsteiner R.W., Ravi H., Joshi S., Guiffre A.C., Sloan C.M., Happe S., Howard S.D. (2013). Advancing genetic testing for deafness with genomic technology. J. Med Genet..

[20] de Brouwer A.P., Pennings R.J., Roeters M., Van Hauwe P., Astuto L.M., Hoefsloot L.H., Huygen P.L., Helm B.v.D., Deutman A.F., Bork J.M. (2003). Mutations in the calcium-binding motifs of CDH23 and the 35delG mutation in GJB2 cause hearing loss in one family. Hum. Genet..

[21] Lerat J., Magdelaine C., Derouault P., Beauvais-Dzugan H., Bieth E., Acket B., Arne-Bes M., Sturtz F., Lia A. (2019). New PRPS1 variant p.(Met68Leu) located in the dimerization area identified in a French CMTX5 patient. Mol. Genet. Genom. Med..

[22] Jerger J. (1970). Clinical Experience With Impedance Audiometry. Arch. Otolaryngol..

[23] Wang H., Chen Y., Lv J., Cheng X., Cao Q., Wang D., Zhang L., Zhu B., Shen M., Xu C. (2024). Bilateral gene therapy in children with autosomal recessive deafness 9: Single-arm trial results. Nat. Med..

[24] Zhang L.-P., Chai Y.-C., Yang T., Wu H. (2013). Identification of novel OTOF compound heterozygous mutations by targeted next-generation sequencing in a Chinese patient with auditory neuropathy spectrum disorder. Int. J. Pediatr. Otorhinolaryngol..

[25] Wu J., Chen J., Ding Z., Fan J., Wang Q., Dai P., Han D. (2023). Outcomes of cochlear implantation in 75 patients with auditory neuropathy. Front. Neurosci..

[26] Tropitzsch A., Schade-Mann T., Gamerdinger P., Dofek S., Schulte B., Schulze M., Fehr S., Biskup S., Haack T.B., Stöbe P. (2023). Variability in Cochlear Implantation Outcomes in a Large German Cohort With a Genetic Etiology of Hearing Loss. Ear Hear..

[27] Kang B., Lu X., Xiong J., Li Y., Zhu J., Cai T. (2022). Identification of four novel variants in the CDH23 gene from four affected families with hearing loss. Front. Genet..

[28] Chen S., Francioli L.C., Goodrich J.K., Collins R.L., Kanai M., Wang Q., Alföldi J., Watts N.A., Vittal C., Gauthier L.D. (2023). A genomic mutational constraint map using variation in 76,156 human genomes. Nature.

[29] Gallo S., Trevisi P., Rigon C., Caserta E., Ali D.S., Bovo R., Martini A., Cassina M. (2021). Auditory Outcome after Cochlear Implantation in Children with DFNB7/11 Caused by Pathogenic Variants in *TMC1* Gene. Audiol. Neurotol..

[30] Lachgar M., Morín M., Villamar M., del Castillo I., Moreno-Pelayo M. (2021). A Novel Truncating Mutation in HOMER2 Causes Nonsyndromic Progressive DFNA68 Hearing Loss in a Spanish Family. Genes.

[31] Santarelli R., Scimemi P., Cama E., Domínguez-Ruiz M., Bonora C., Gallo C., Rodríguez-Ballesteros M., del Castillo I. (2023). Preservation of Distortion Product Otoacoustic Emissions in OTOF-Related Hearing Impairment. Ear Hear..

[32] Belova V., Pavlova A., Afasizhev R., Moskalenko V., Korzhanova M., Krivoy A., Cheranev V., Nikashin B., Bulusheva I., Rebrikov D. (2022). System analysis of the sequencing quality of human whole exome samples on BGI NGS platform. Sci. Rep..

[33] Andrews S. (2010). FastQC: A Quality Control Tool for High Throughput Sequence Data. http://www.bioinformatics.babraham.ac.uk/projects/fastqc/.

[34] Joint Genome Institute. https://jgi.doe.gov/data-and-tools/software-tools/bbtools/bb-tools-user-guide/bbduk-guide/.

[35] Li H., Durbin R. (2009). Fast and accurate short read alignment with Burrows—Wheeler transform. Bioinformatics.

[36] Li H., Handsaker B., Wysoker A., Fennell T., Ruan J., Homer N., Marth G., Abecasis G., Durbin R. (2009). 1000 Genome Project Data Processing Subgroup. The Sequence Alignment/Map format and SAMtools. Bioinformatics.

[37] Picard Toolkit (2019). version 2.22.4.

[38] Li H. (2011). A statistical framework for SNP calling, mutation discovery, association mapping and population genetical parameter estimation from sequencing data. Bioinformatics.

[39] Poplin R., Chang P.-C., Alexander D., Schwartz S., Colthurst T., Ku A., Newburger D., Dijamco J., Nguyen N., Afshar P.T. (2018). A universal SNP and small-indel variant caller using deep neural networks. Nat. Biotechnol..

[40] Wang K., Li M., Hakonarson H. (2010). ANNOVAR: Functional annotation of genetic variants from high-throughput sequencing data. Nucleic Acids Res..

[41] Li Q., Wang K. (2017). InterVar: Clinical Interpretation of Genetic Variants by the 2015 ACMG-AMP Guidelines. Am. J. Hum. Genet..

[42] Talevich E., Shain A.H., Botton T., Bastian B.C. (2016). CNVkit: Genome-Wide Copy Number Detection and Visualization from Targeted DNA Sequencing. PLOS Comput. Biol..

[43] Gurbich T.A., Ilinsky V.V. (2020). ClassifyCNV: A tool for clinical annotation of copy-number variants. Sci. Rep..

[44] Geoffroy V., Herenger Y., Kress A., Stoetzel C., Piton A., Dollfus H., Muller J. (2018). AnnotSV: An integrated tool for structural variations annotation. Bioinformatics.

[45] Ewels P., Magnusson M., Lundin S., Käller M. (2016). MultiQC: Summarize analysis results for multiple tools and samples in a single report. Bioinformatics.

[46] Köhler S., Gargano M., Matentzoglu N., Carmody L.C., Lewis-Smith D., Vasilevsky N.A., Danis D., Balagura G., Baynam G., Brower A.M. (2020). The Human Phenotype Ontology in 2021. Nucleic Acids Res..

[47] Richards S., Aziz N., Bale S., Bick D., Das S., Gastier-Foster J., Grody W.W., Hegde M., Lyon E., Spector E. (2015). Standards and guidelines for the interpretation of sequence variants: A joint consensus recommendation of the American College of Medical Genetics and Genomics and the Association for Molecular Pathology. Genetics in Medicine.

[48] Barbitoff Y.A., Khmelkova D.N., Pomerantseva E.A., Slepchenkov A.V., Zubashenko N.A., Mironova I.V., Kaimonov V.S., Polev D.E., Tsay V.V., Glotov A.S. (2024). Expanding the Russian allele frequency reference via cross-laboratory data integration: Insights from 7,452 exome samples. Natl. Sci. Rev..

