# Supplementary material for: Heterogeneous Group of Genetically Determined Auditory Neuropathy Spectrum Disorders
Source: Int J Mol Sci. 2024 Nov 22;25(23):12554. doi: 10.3390/ijms252312554 (PMC11641461; doi:10.3390/ijms252312554)
Supplement: Supplementary file 1 [file ijms-25-12554-s001.zip › Document S1. The screening survey (younger than 18 years).pdf]

Screening Diagnostic Questionnaire (for children)

Full Name ____

Age (in full years) ___________________________________

Duration of use of the rehabilitation device: _______________________

Educational segment (underline the correct one): not organized (home), general daycare, general school, special school for hearing-impaired, university, vocational institution.

Mark the correct answer.		**Yes (0 points)**	**No (2 points)**	**Sometimes (1 point)**
**1.**	Does your child have difficulties in communication (with CI/HA)?			
**2.**	Does your child feel uncomfortable wearing a rehabilitation device?			
**3.**	Does your child experience headaches when wearing CI or HA for a long time?			
**4.**	Does your child have difficulties talking on the phone with CI/HA (if using a phone)?			

Mark the correct answer in the corresponding cell.		**Yes (2 points)**	**No (0 points)**
**5.**	Does your child undergo rehabilitation courses?		
**6.**	Does your child study with a speech therapists/deaf teacher?		

Please answer the following questions.

7. What time per a day does your child explore CI or HA?

____

8. How often does your child visit an audiologist?

____

9. ***(Rate on a 10-point scale where 0 – no change and 10 – life has become brighter and more fulfilling)***

Your child's life has changed since they started wearing HA or CI.

0 1 2 3 4 5 6 7 8 9 10
